# Effects of crystalline glucocorticoid triamcinolone acetonide on cultered human supraspinatus tendon cells

**DOI:** 10.3109/17453670902988360

**Published:** 2009-06-01

**Authors:** Herbert Tempfer, Renate Gehwolf, Christine Lehner, Andrea Wagner, Maia Mtsariashvili, Hans-Christian Bauer, Herbert Resch, Mark Tauber

**Affiliations:** ^1^Department of Organismic Biology, Division of Zoology and Functional Anatomy, University of SalzburgSalzburgAustria; ^2^Applied Cell Bioloogy and Developmental Biology Unit, Paracelsus Private Medical UniversitySalzburgAustria; ^3^Department of Traumatology and Sports Injuries, University Hospital of Salzburg HT and RG contributed equally to this Salzburg paperSalzburgAustria

## Abstract

**Background** Rotator cuff tears are a common cause of shoulder pain and impairment. Subacromial glucocorticoid injections are widely used for treatment of epiphenomenons of chronic impingement syndrome with the possible side effects of tendon rupture and impaired tendon healing.

**Methods** Using qRT-PCR, western blot, immunoflourescence, and measurement of ^3^H-thymidine uptake we investigated the effects of the crystalline glucocorticoid triamcinolone acetonide (TAA) when added to the culture medium of isolated human rotator cuff tendon cells.

**Results** After 2 weeks of incubation, the cells had lost their fibroblastic appearance and parallel orientation, which is characteristic of cellular degeneration in vivo. Moreover, expression and secretion of collagen I was strongly reduced, and there was a decrease in proliferation rate. Cell migration was blocked and the rate of expression of the matrix metalloproteinases MMP2, MMP8, MMP9, and MMP13 was reduced, but expression of TIMP1 (a tissue inhibitor of MMPs) was upregulated, indicating a reduction in the cellular capacity for tendon repair. In addition, changes in cellular differentiation were observed: the number of adipocytes increased and levels of the protein Sox9—a marker of differentiating and mature chondrocytes—were elevated in triamcinolone acetonide treated cells.

**Interpretation** These results may indicate that the use of TAA is one reason for weaker mechanical tendon properties and for the high rate of re-rupture after supraspinatus tendon repair.

## Introduction

Rotator cuff tears causing shoulder pain and disability are common and may be caused by trauma or chronic degenerative processes. Subacromial injection of long-acting corticosteroids is a common treatment to relieve shoulder pain and inflammation. The short-term effect is satisfactory (McInerey et al. 2006). However, some studies have suggested that there can be impairment of mechanical tendon properties and a high rate of tendon rupture after long-term treatment (Hugate et al. 2004, Nichols 2005). When glucocorticoids are injected into an intact bursa, no direct contact between the corticoid crystals and the tendon cells is to be expected. If the supraspinatus tendon is at least partially torn or the bursa is damaged, however, it is likely that there will be contact between the corticoid and cells—with possibly negative side effects. Moreover, injection experiments in cadavers have revealed an inaccuracy rate of up to 40% for intrabursal injection of substances directly into the tendon (Mathews and Glousman 2005).

Several reports have described the effects of glucocorticoids on tendon cells and chondrocyte cultures. Scutt et al. (2006) showed that dexamethasone treatment inhibits cell proliferation and reduces collagen synthesis in primary rat tail tendon cells. In addition, an inhibitory effect of glucocorticoids on tendon cell proliferation and proteoglycan production has been found in vitro and in vivo (Wong et al. 2004).

Most studies on the effects of corticosteroids in in vitro systems have used the lipoid corticosteroid dexamethasone (Scutt et al. 2006). It has been shown that triamiconolone acetonide (TAA) reduces proteoglycane synthesis and that dexamethasone inhibits the migration of tendon cells (Tsai et al. 2003, Wong et al. 2005).

In this paper, we show for the first time the effects of TAA on cell proliferation, collagen synthesis and secretion, production of matrix remodeling molecules, and differentiation status in primary cultures of human supraspinatus tendon cells.

## Material and methods

### Materials

DMEM, collagenase, and fetal calf serum were obtained from Gibco/Invitrogen (Lofer, Austria). Cell culture plastic material was obtained from Nunc (Roskilde, Denmark). All other chemicals were purchased from Sigma (Vienna, Austria). For triamcinolone acetonide treatment, the brand product Volon A40 (Dermapharm GmbH, Vienna, Austria) was used.

The antibodies used for immunohistochemistry were anti-Sox9 (rabbit polyclonal sc-20095; Santa Cruz Biotechnology, santa Cruz, CA) and anti-collagen I (rabbit polyclonal ab292; Abcam, Cambridge, UK).

### Isolation and culture of tendon-derived cells

Supraspinatus tendon cells (STCs) were isolated from biopsies of intact human supraspinatus tendons, which were obtained during posttraumatic surgical interventions not involving the rotator cuff (open Bankart repair in 3 patients, open glenoid fracture fixation in 4 patients) with informed consent from the patients (3 males aged 15, 35, and 40, and 4 females aged 39, 43, 45, and 56).

The biopsies (weighing about 0.5 g each) were cut into small pieces under sterile conditions, followed by a 4-h digestion in DMEM supplemented with 30 mg/mL collagenase II (Gibco) at 37°C, 95% humidity, and 5% CO_2_. After digestion, the cells were pelleted, washed in PBS, and subsequently cultured in 25-cm^2^ cell culture flasks with DMEM supplemented with 10% fetal bovine serum (FBS). The method described yields 50 cm^2^ of subconfluent STCs after 1 week of culture. Sub-confluent cells were incubated with DMEM, 10% FBS, and 1 mg/mL TAA for 2 weeks. For the proliferation assay, the cells were incubated for only 1 week. Control cells were incubated with DMEM and 10% FBS for the same periods of time.

The cultures obtained from each single patient were treated separately. All experiments described were performed either with primary cultures of tendon cells or with cells passaged once.

### Cell characterization

Adipocytes were stained with a 0.5% Sudan III solution for 30 min at 37°C, and the proportion of positive cells was determined. Chondrocytes were identified by western blot using anti-Sox9 antibody. Sox9 has been reported to be a potent activator of type II collagen expression and is commonly used as a phenotypic marker of articular chondrocytes (Bi et al. 1999). In addition, accumulation of mucopolysaccharides associated with chondrocyte differentiation was shown by Alcian blue staining (Phornphutkul et al. 2006).

For immunofluorescence, STCs grown on coverslips were fixed for 5 min in 100% methanol at –20°C. The cells were washed 3 times with phosphate-buffered saline and were permeabilized for 10 min in 0.25% Triton X-100 in phosphate-buffered saline, followed by three washing steps. After blocking for 30 min in 5% BSA in phosphate-buffered saline at room temperature, the fixed cells were incubated overnight with rabbit anti-Sox9 antibody (diluted 1:100 in blocking solution) at 4°C. After 3 washes, the cells were incubated with secondary antibody (Alexa Fluor 568 goat anti-rabbit IgG diluted 1:2,000 in blocking solution) for 1 h at 37°C. After 3 final washing steps, the coverslips were mounted with Mowiol 4-88 (Calbiochem). Images were taken using a Zeiss Axio-plan microscope equipped with the Zeiss AxioVision imaging system.

### Determination of collagen I secretion western blotting and proliferation assay

The measurement of collagen I secretion was done as described by Yang et al. (2004). Briefly, 1 mL of cell supernatant was digested with 100 µg/mL pepsin under acidic conditions, precipitated with 3M NaCl, dialyzed against 0.05 M NH_4_CO_3_, and vacuum dried.

For western blot, proteins in the supernatant were separated by polyacrylamide gel electrophoresis (SDS-PAGE). For each sample, equal amounts of protein were loaded. The protein content of lyzed cells was measured with the BCA protein assay kit (Pierce). Blotting was performed as previously described by Yang et al. (2004) using the rabbit polyclonal antibody directed against collagen I (ab292; Abcam).

Proliferation was measured with the ^3^H-thymidine incorporation assay. Cells were seeded in 24-well plates at a density of 5 × 10^5^ cells per well and incubated with triamcinolone acetonide (TAA). The controls were not treated with TAA. After 24 h, 48 h, and 96 h of incubation with 1 µCi ^3^H-thymidine for 6 h each, the cells were harvested, lysed in 1 M NaOH, and the radioactivity measured in a β-scintillation counter (Beckman). All reading points for each patient were done in triplicate. We used counts per minute per mg protein as a measure of cell proliferation, and background counts were subtracted.

### RNA preparation and quantitative real-time (qRT-) PCR

Total RNA was isolated using the PureZol RNA isolation kit (BioRad). RNA yield and integrity were evaluated spectrophotometrically and by agarose gel electrophoresis. Prior to first-strand cDNA synthesis, 1 µg total RNA was digested with DNase I (Fermentas, St. Leon-Roth, Germany). Reverse transcription was performed according to Gehwolf et al. (2002). Primers for qRT-PCR were designed to be intron spanning according to the annotated human sequences of MMP2, MMP9, TIMP1, GAPDH, and HPRT, respectively (Table). The PCR was carried out in a Stratagene MX3000 real-time PCR cycler using the dsDNA-binding fluorescent dye SYBR-green I (Invitrogen) in a reaction volume of 50 µL.

**Table:** Primer sequences for RT-PCR and quantitative RT-PCR

Gene product	Forward primer sequence	Reverse primer sequence	Accession no.
MMP1	5´-TAGAACTGTGAAGCATATCGATG-3´	5´-AGTTGAACCAGCTATTAGCTTTC-3´	NM_002421
MMP2	5´-TCTACTCAGCCAGCACCCTGGA-3	5´-TGCAGGTCCACGACGGCATCCA-3´	NM_004530.2
MMP3	5´-TACTGGAGATTTGATGAGAAGAG-3´	5´-TACAGATTCACGCTCAAGTTCC-3´	NM_002422
MMP8	5´-TCAGGTGCCTTTCCAGGAATAG-3´	5´-TACAGTGATGGGAAACAATGAC-3´	NM_002424
MMP9	5´-TTCGACGTGAAGGCGCAGATGGT-3´	5´-TAGGTCACGTAGCCCACTTGGTC-3´	NM_004994.2
MMP13	5´-TGCAGCTGTTCACTTTGAGGA -3´	5´-TGGCATGACGCGAACAATACG-3´	NM_002427
MMP14	5´-TACCGACAAGATTGATGCTGCTC-3´	5´-TCTACCTTCAGCTTCTGGTTG-3´	NM_004995
TIMP1	5´-TGGACTCTTGCACATCACTACCTGC-3´	5´-AGGCAAGGTGACGGGACTGGAA-3´	NM_003254.2
GAPDH	5´-AACATCATCCCTGCCTCTAC-3´	5´-CTGCTTCACCACCTTCTTG-3´	NM_002046
HPRT	5´-TGCTTTCCTTGGTCAGGCAGTATA-3´	5´-GCGATGTCAATAGGACTCCAGAT-3´	NM_000194

All samples were analysed in triplicate. A single reaction contained 1 µL cDNA, 1 unit TrueStart Taq DNA polymerase (Fermentas), 1 × Taq DNA polymerase reaction buffer (Fermentas), 3.5 mM MgCl_2_, each dNTP at 0.2 mM, each of the primers at 300 nM, and 0.1 × SYBR-green I. The thermal cycling program consisted of an initial denaturation step of 3 min at 95°C followed by 40 cycles of 30 seconds at 95°C, 30 seconds at 58°C, and 30 seconds at 72°C. Melting curve analysis and agarose gel electrophoresis were performed to ensure that a single PCR product had been amplified. Reactions without template were included as negative controls. Transcript levels were normalized to the expression of GAPDH and HPRT mRNAs. Non-quantitative RT-PCR amplification of MMP1, MMP3, MMP8, MMP13, and MMP14 was performed with the same PCR reaction conditions but without SYBR-green. PCR products were analyzed using agarose gel electrophoresis and visualized by ethidium bromide staining.

### Gelatin zymography

Gelatine zymography was performed as described previously (Krizbai, et al. 2000). Briefly, cells were lysed in a buffer containing 20 mM Tris-HCl (pH 7.5), 150 mM NaCl, 1% Triton X-100, and 0.1% SDS. Equal amounts of protein were loaded on 7.5% polyacrylamide gels containing casein (1 mg/mL). Following electrophoresis, the gels were given two 10-min washes with ddH_2_O containing 2.5% Triton X-100, then incubated with a substrate buffer (50 mM Tris-HCl (pH 8.0) containing 5 mM CaCl_2_). The gels were then stained with Coomassie blue for 15 min followed by several washing steps.

### Wounding assay

After cells cultured on collagen-coated coverslips had reached confluence, the cell layers were wounded with a razor blade. The coverslips were then rinsed with PBS and cultured for another 24 h, either with TAA or without (controls). Any cells migrating from the wound edge into the denuded area were observed and documented.

### Statistics

In all graphs, means and standard deviations are given. Student’s t-test was used to test for effects of TAA treatment, and the significance level was set at p = 0.05.

## Results

**Figure 1.**
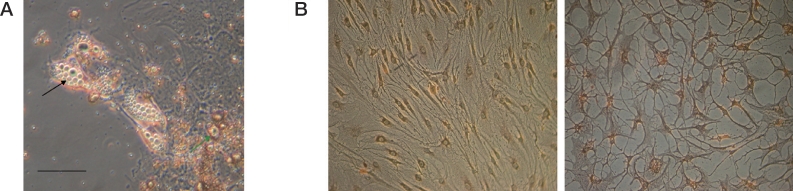
A. Clusters of differentiated adipocytes with typical lipid droplets in cells (black arrow) after treatment with triamicolone acetonide. The green arrow indicates triamicolone acetonide crystals. B. Untreated cells show a spindle-shaped phenotype typical of tendon cells, whereas treated cells totally lose their orientation (right panel). * p < 0.001.

Already after 1 week, the number of adipocytes identified increased. After 2 weeks of incubation with TAA, a 20-fold increase in adipocytes was observed compared to untreated controls (Figure 1). The total number of adipocytes did not exceed 0.4% of all treated cells.

The adipocytes usually appeared in groups of 3–4 cells— in contrast to the spontaneously differentiated control cells, which only showed single adipocytes (Figure 1). Generally, these groups of cells were found to have direct physical contact with crystalline clusters of TAA.

**Figure 2.**
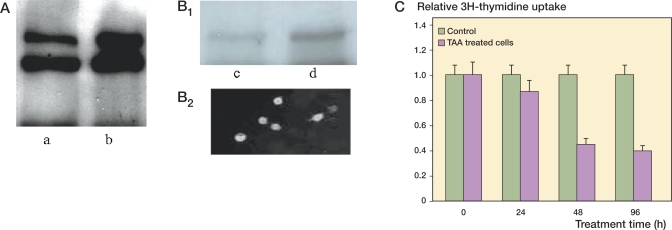
A. Downregulation of collagen type I secretion in TAA-treated supraspinatus tendon cells (STCs) (a: treated cells; b: control). The two separate bands seen in blot A are α1 chain and α2 chain of collagen type I, respectively. B. Sox9 is upregulated in treated cells (d) relative to control cells (c). C. TAA-treated cells show a decrease in 3H-thymidine uptake depending on the duration of TAA treatment. D. After TAA treatment, several cells stained positively for Sox9, whereas no positive cells were found in the control (not shown). * p < 0.001.

Western blot analysis showed upregulation of Sox9 in TAA-treated cells (Figure 2). Secretion of collagen type I was reduced. Staining with Alcian blue showed positive cells in the treatment group, whereas no stained cells were found in the controls. Of 10,000 cells counted, 89 positive cells were found.

Cell proliferation was significantly reduced as a result of treatment with TAA, by a factor of 3 compared to untreated cells within 96 hours (Figure 2).

**Figure 3.**
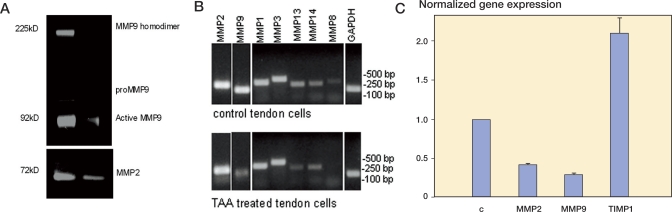
A. Results of gelatin zymography showing the downregulation of MMP2 and MMP9 by triamcinolone acetonide (right lane) compared to untreated control cells (left lane). RT-PCR result showing downregulation of MMP2, MMP8, MMP9, and MMP13 mRNA levels after TAA treatment. B. Levels of MMP1, MMP3, and MMP14 mRNA were unchanged. C. qRT-PCR showed significant downregulation of MMP2 and MMP9 mRNA levels, and significant upregulation of TIMP1 mRNA. Gene expression levels of MMP2, MMP9, and TIMP1 were normalized to the expression of housekeeping genes GAPDH and HPRT. * p < 0.001.

In qRT-PCR applications, expression of MMP2 and MMP9 mRNA was found to be significantly downregulated by TAA treatment—by 58% (SD 0.9) for MMP2 and by 72% (SD 1.9) for MMP9—whereas the expression of TIMP1 mRNA was upregulated by 111% (SD 21). The decrease in MMP2 and MMP9 levels was confirmed by gelatine zymography. In the case of MMP9, all the functional forms of the enzyme that have been described to date (active MMP9 with a size of 92 kD, pro-MMP9 of about 130 kD, and the homodimer of 225 kD) were downregulated (Figure 3).

RT-PCR revealed downregulation of MMP8 and MMP13 mRNAs after TAA treatment; no changes in the levels of MMP1, MMP3, and MMP14 mRNAs were found (Figure 3).

**Figure 4.**
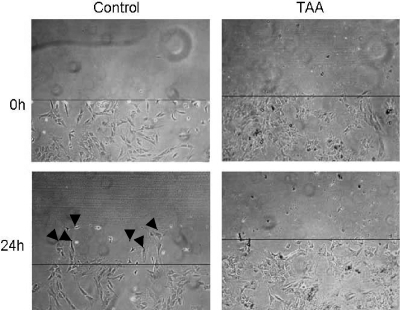
Within 24 h, TAA had totally inhibited cell migration whereas untreated control cells could apparently invade the denuded area (arrowheads).

The wounding assay showed total blockage of cell migration in TAA-treated cells, whereas the control cells actively migrated into the wounded area (Figure 4).

The TAA product used (with brand name Volon A40) contains the preservative phenyl carbinol at a concentration of 40 mg/mL. No effects were observed in cells incubated with phenyl carbinol at a concentration of 40 mg/mL.

## Discussion

We focused our investigations on the supraspinatus tendon, the most frequently affected tendon in rotator cuff injuries. For this study, we only used tendons with no signs of degeneration. We hypothesized that any observed degeneration caused by TAA on healthy tendon would be likely to aggravate impairment on an initially damaged tendon. Moreover, contact between TAA and intact tendon material appears to occur surprisingly often, since up to 40% of all intrabursal injections are reported to come in contact with intact tendons, and thus with tendon cells (Mathews and Glousman 2005).

We used an in vitro model of cultured tendon cells because it is the only choice to study the immediate effects of TAA on cells during a short period of time. In our experiments only one concentration of TAA was used, which is similar to that to which the tendon cells in tissue are exposed. Due to the poor solubility of TAA in culture media and buffer solutions, dose-dependent effects are difficult to interpret. Also, due to the chemical nature of TAA, the substance is slowly dissolving in aqueous solution but it is slowly diffusing in tissue or cellular layers. While the use of organic solvents would increase solubility in culture, they would not reflect the in vivo situation.

We found that TAA caused a decrease in proliferation rates and collagen synthesis, and also triggered a differentiation process resulting in an increased number of adipocytes and chondrocytes after 2 weeks in culture. Moreover, TAA inhibited tendon cell migration by downregulating the expression of matrix metalloproteinases MMP2 and MMP9 with concurrent upregulation of TIMP1.

Corticosteroids have a directly inhibitory effect on the transcription of MMP9, which may be one reason for the effects observed (Van den Steen et al. 2002). Moreover, hydrocortisone has been shown to inhibit transcription of both the MMP2 and MMP9 genes, by influencing the binding activity of activating protein 1 (AP1) (Aljada et al. 2001).

We chose MMP2 and MMP9 for this study because of their essential role in tendon cell migration, which is likely to be important for tendon healing. Moreover, they are also key players in neovascularization of renewed tissue (Ragoowansi et al. 2003). Thus, it is feasible that any alteration in MMP2 and MMP9 levels and their relative ratios to TIMP1 may affect the healing potential of injured or freshly reconstructed tendon tissue.

Generally, MMPs and TIMPs are reported to play a major role in tendon diseases and in tendon healing. Expression of MMP13, for example, has been shown to be elevated in torn rotator cuff tendons (Lo et al. 2004).

Downregulation of collagen type I synthesis in tendon-derived cells caused by dexamethasone has already been described (Scutt et al. 2006). Our data show that it was not only the amount of collagen type I that was reduced by the glucocorticoid TAA: the level of secretion of collagen type I (which is known to be important for tendon stability) was affected (Kannus 2000).

One reason for the reduced rate of collagen type I secretion could be the change in terminal differentiation of tendon cells caused by TAA, as suggested by our data. However, the number of newly formed adipocytes and chondrocytes after incubation of tendon cell cultures with TAA was too low to explain this decrease. The fact that glucocorticoids give rise to other cells in culture is interesting. In vivo, these differentiation processes may act over a longer time period with so far unknown effects on tendon strength.

As a third cellular parameter, we chose cell proliferation as a response to corticoid treatment. The observed reduction in proliferation may possibly be associated with a reduced intra-cellular level of TGF-b, which has been shown to be caused by triamcinolone (Carroll et al. 2002). Generally, mature tendon cells have very low proliferative capacity with a proliferation index around 1% (Matsuda 1994). However, under conditions of culture a small population of these cells seems to be induced to proliferate (Scutt et al. 2006). Conceivably, this population plays a major role in tendon healing, not least because of the stem cell properties attributed to some tendon-derived cells (Salingcarnboriboon et al. 2003, Bi et al. 2007). Thus, the deceleration of proliferation may contribute to the impaired tendon healing described by Nichols (2005) as a negative side effect of local steroid treatment in ruptured supraspinatus tendon. Reduced matrix turnover and cell migration are further possible reasons for the weakening of the tendon’s mechanical properties, and could be jointly responsible for the high failure rate reported after rotator cuff repair (Jost et al. 2000, Mellado et al. 2006). Watson (1985) reported a poorer outcome in a series of 89 cases after rotator cuff repair in patients with more than 4 preoperative local steroid injections. A correlation was seen between a higher number of steroid injections and a softer residual cuff, and it is assumed that poor results were due to this soft tissue (Matsuda 1994).

Based on our findings, for therapeutic purposes cautious application of crystalline TAA is reasonable, especially in cases with already diagnosed rotator cuff tears and subsequent cuff repair. This study also emphasizes the importance of accurate intrabursal injection. Finally, future studies will show whether the effects of crystalline TAA observed here can also be seen in other tendon tissue.
